# The use of plant extracts and their phytochemicals for control of toxigenic fungi and mycotoxins

**DOI:** 10.1016/j.heliyon.2020.e05291

**Published:** 2020-10-22

**Authors:** Rhulani Makhuvele, Kayleen Naidu, Sefater Gbashi, Velaphi C. Thipe, Oluwafemi A. Adebo, Patrick B. Njobeh

**Affiliations:** a Department of Biotechnology and Food Technology, Faculty of Science, University of Johannesburg, Doornfontein Campus, P.O. Box 17011, Doornfontein Campus, Gauteng, 2028, South Africa; b Laboratório de Ecotoxicologia - Centro de Química e Meio Ambiente - Instituto de Pesquisas Energéticas e Nucleares (IPEN) - Comissão Nacional de Energia Nuclear- IPEN/CNEN-SP, Av. Lineu Prestes, 2242 - Butantã, 05508-000, São Paulo, Brazil

**Keywords:** Food science, Food safety, Mycotoxin, Fungal invasion, Detoxification, Plant extracts, Nanosponge, Food and feed

## Abstract

Mycotoxins present a great concern to food safety and security due to their adverse health and socio-economic impacts. The necessity to formulate novel strategies that can mitigate the economic and health effects associated with mycotoxin contamination of food and feed commodities without any impact on public health, quality and nutritional value of food and feed, economy and trade industry become imperative. Various strategies have been adopted to mitigate mycotoxin contamination but often fall short of the required efficacy. One of the promising approaches is the use of bioactive plant components/metabolites synergistically with mycotoxin-absorbing components in order to limit exposure to these toxins and associated negative health effects. In particular, is the fabrication of β-cyclodextrin-based nanosponges encapsulated with bioactive compounds of plant origin to inhibit toxigenic fungi and decontaminate mycotoxins in food and feed without leaving any health and environmental hazard to the consumers. The present paper reviews the use of botanicals extracts and their phytochemicals coupled with β-cyclodextrin-based nanosponge technology to inhibit toxigenic fungal invasion and detoxify mycotoxins.

## 1 Introduction

Food and feed contamination by toxigenic fungi is of serious health concern and has been recorded since mankind initiated the cultivation and storage of agricultural commodities. These toxigenic fungi belong mainly to the genera *Aspergillus, Fusarium*, *Claviceps, Penicillium, Stachybotrys,* and *Altenaria,* etc. They produce toxic substances, some of which are referred to as mycotoxins. Cumulative evidence from the literature suggest that these mycotoxins do not present any apparent biochemical importance in fungal development or growth. However some secondary metabolites play a role in virulence, development and pathogenicity (Perincherry et al., 2019; Venkatesh and Keller, 2019). Mycotoxins have adverse effects on humans and animals even at low concentrations (Vila- Donat et al., 2018; Adekoya et al., 2019), and the proliferation of toxigenic fungi on food and feed is always favoured by certain environmental factors such as humidity and temperature, as well as certain biotic conditions (*i.e.* substrate composition). High incidences of mycotoxin occurrence are usually reported in tropical regions such as Asia and sub-Saharan Africa, where conditions for their proliferation are optimal. Mycotoxins contaminate mainly commodities such as cereals, grains, nuts, and their by-products during production, pre-harvest and post-harvest (Milicevic et al., 2010; Zain, 2011; Nleya et al., 2018; Balendres et al., 2019; Gbashi et al., 2019). These toxins often enter the body mainly via ingestion, as well as by inhalation, parental and dermal exposure routes. The toxins can also enter the food chain through infected crops which are consumed either directly or indirectly by humans or animals as feed sources. As such, they appear in meat, milk and eggs (Hojnik et al., 2017).

Mycotoxin contamination is of serious concern for food safety and security worldwide. These toxins account for enormous economic losses in agricultural productivity and trade, with more severity in poor and developing countries. It is estimated that about 60–80% of crops worldwide can be contaminated by mycotoxins, thus resulting in significant economic losses (Eskola et al., 2019). The toxic effects of mycotoxins are persistent, and they are difficult to completely eradicate once they enter the food/feed chain. In the agricultural industry, mycotoxins cause loss in livestock production due to reduced growth rates, decreased immunity and fertility, reduced eggs, meat and milk production, and increased mortality (Thipe et al., 2020). Furthermore, the effects associated with mycotoxins cause veterinary and healthcare costs (Marroquin-Cardona et al., 2014; Kagot et al., 2019). This contamination of food and feedstuffs by mycotoxins equally reduces the nutritional value, quality and safety of food and feed (Luo et al., 2018). Many countries have established regulatory limits on mycotoxins in agricultural commodities to limit human and animal health risks associated with them (Marroquin-Cardona et al., 2014; Haque et al., 2020). Mycotoxins cause a disease known as mycotoxicosis (Bennett and Klich, 2003; Liew and Mohd-Redzwan, 2018). The health effects of mycotoxins are extensive as they potentiate some hepatotoxic, nephrotoxic, mutagenic, genotoxic, carcinogenic, immunosuppressive and teratogenic properties. The most prevalent mycotoxins of agricultural importance include the aflatoxins (AFs), ochratoxins (OTA), fumonisins (FBs), trichothecenes and zearalenone (ZEN). These have been given serious attention due to the health risks that they pose in humans and animals (Dikhoba et al., 2019; Celik, 2020).

Various approaches to control and prevent mycotoxins in food and feed have been developed. These approaches are categorised as chemical and micro-biological methods (Adebo et al., 2017; Adebiyi et al., 2019). These methods were shown to be effective in the prevention of growth of toxigenic fungi and production of associated mycotoxins during pre-harvest, after harvest food and during storage of food commodities. Chemical methods involve the use of chemicals such as ammonia, sodium hydroxide, hydrochloric acid, butylated hydroxytoluene, butylated hydoxyxyanisole and oltipraz to decontaminate mycotoxins (Galvano et al., 2001; Karlovsky et al., 2016; Čolović et al., 2019). In addition to their inefficacy in decontamination of mycotoxins, their long-term extreme use is still limited due to their residual toxic product/s, public health and environmental concerns (Abdel-Fattah et al., 2018; Alberts et al., 2019; Sadhasivam et al., 2019; Meng et al., 2020) coupled with the interference with nutrients and organoleptic properties of food and feed (Celik, 2020).

Physical methods include cleaning, dehulling, sorting, milling and ultra-violet light, pulsed light, cold plasma as well as irradiation. Other physical methods involve the use of adsorbents or binders such as activated charcoal, bentonite, zeolites and sepiolite clay. The methods have been effectively applied in decontaminating mycotoxins. However, technology implementation costs, potential residual toxic effects, poor adsorption and low specificity (i.e. selective action) against some mycotoxins still hamper their routine application (Mahato et al., 2019; Celik, 2020). Microbiological methods entail the use of probiotic bacteria, yeasts and their enzymes, which are effective in reducing mycotoxins in food and feed (Tian et al., 2016; Adebo et al., 2017; Udomkun et al., 2017). However, these microorganisms and their enzymes often interfere with nutrient supply resulting in undesirable products. Apart from this, enzymatic degradation by-products still limit their uses (Juodeikiene et al., 2012; Adebiyi et al., 2019; Lyagin and Efremenko, 2019). Therefore, there is a need to discover alternative methods that can prevent fungal colonization of agricultural commodities, detoxify or bio-transform mycotoxin residues to less- or non-toxic forms without any limitations (Iram et al., 2015; Powers et al., 2019; Haque et al., 2020).

Botanicals such as essential oils, spices, herbs and crude extracts present outstanding alternatives for the discovery of biofungicides and nutraceuticals for mitigating mycotoxicosis and related infections. Botanicals are generally regarded as environmentally friendly and safer alternative sources of bioagents for the control of fungi and mycotoxins in food and feed (Iram et al., 2016; Adebo et al., 2020; Prakash et al., 2020). They are more affordable as opposed to other materials used for the same purpose, they provide a synergistic approach as protectants of fungal/mycotoxin contamination and further stimulate pathways that elicit the natural defence systems in plant tissues (da Cruz Cabral et al., 2013; Alberts et al., 2019; Gacem et al., 2020; Meng et al., 2020). They contain various phytochemicals with pharmacological properties against various diseases. Recent studies have been conducted on the possible application of botanicals as bio-fungicides and nutraceuticals to ameliorate the proliferation of toxigenic fungi and mycotoxin contamination in food and feed (Dikhoba et al., 2019; Ponzilacqua et al., 2019; Kavitha et al., 2020). This review aimed to elucidate the use of botanical extracts and their phytochemical compounds to prevent and detoxify mycotoxins without any adverse effect on the nutritional value of food and feed. We also review nano-encapsulation technology which can be adopted to improve bioavailability and solubility of phytochemicals used as biofungicides and nutraceuticals to prevent growth of mycotoxigenic fungi and subsequent mycotoxin contamination.

## 2 Mitigation of toxigenic fungi and mycotoxins using botanical methods

### 2.1 Importance of botanicals

Plants have been widely used in folk medicine since time immemorial in order to treat and prevent various ailments from one generation to another (Horn and Vargas, 2008; Street and Prinsloo, 2013). Despite the advancement made in modern medicine, many populated groups in developing countries still depend on traditional medicine for preventing and treating various ailments. This is due to cultural beliefs, low cost and effectiveness (Moura-Costa et al., 2012; Ayele, 2018). According to the World Health Organization (WHO, 2001), approximately 80% of the population of the world still depend on traditional medicine for primary healthcare (Prakash et al., 2020). Recent studies have also created renewed interest in the use of botanicals and their compounds as nutraceuticals in this regard in both developed and developing countries (Galvano et al., 2001; Reddy et al., 2010; Anjorin et al., 2013; Dikhoba et al., 2019). The advantage of using plants for drug discovery is due to their abundance in nature and wide distribution geographically. A considerable number of drugs have been synthesized from plants based on their use in traditional medicine (Van Wyk et al., 1997; Dias et al., 2012). Africa is richly endowed with a wealth of medicinal plants. However, very few studies have looked into exploiting their constituents for mycotoxin detoxification (Stoev et al., 2019; Makhuvele et al. unpublished data), and thus necessitate studies in this regard. The following sections provide an overview of various botanicals that exhibit antifungal activities. In addition, we also follow a nanoencapsulation approach through the fabrication and use of nanosponges to improve the stability from oxidation/degradation, availability, and demonstrate the most pronounced efficacy of botanicals.

### 2.2 Phytochemicals as a source of therapeutics and nutraceuticals

Plants produce secondary metabolites as a defence mechanism against pathogenic microorganisms, insects and adverse environmental conditions. These metabolites are known as phytochemicals, which are non-nutritive (Prakash et al., 2020) and to some extent essential oils. However, they can protect humans and animals against certain diseases caused by microorganisms or toxins associated with them due to the antimicrobial properties they possess (Palombo, 2011; Shin and Park, 2018; Redondo-Blanco et al., 2019). The metabolites are the most promising chemo-preventative agents for future drug discovery and development (Alabi et al., 2011). There are various major groups of phytochemical compounds discovered to date and differ according to their chemical structures (Das et al., 2020). These major groups include phenolic compounds, flavonoids, phytosterols, carotenoids, tocols, terpenoids, alkaloids, saponins, tannins, aromatic acids, glucosinolates, carotenoids, essential oils, chlorophyll and organic acids as well as proteases inhibitors (Bhattachar, 2011; Adebo and Gabriela Medina-Meza, 2020; Loi et al., 2020). Such compounds may act directly or indirectly to protect against ailments or pathogens, because they contain antimutagenic, antigenotoxic, antimicrobial, anthelmintic, anticarcinogenic, antiproliferative and anti-inflammatory as well as antioxidant properties (Galvano et al., 2001; Makhafola et al., 2017; Velu et al., 2018; Lahlou et al., 2019).

### 2.3 Antifungal effects and detoxification abilities of plant extracts and their phytochemicals compounds on toxigenic fungi and mycotoxin toxicity

Plants possess antimutagens, antimicrobial, antioxidants or anticarcinogens capable of compromising the toxic and genotoxic effects of mycotoxins (Madrigal-Santillan et al., 2010; Anjorin et al., 2013; Powers et al., 2019). Antioxidants protect the cell membranes and macromolecules by scavenging free radicals (Wu et al., 2017a). Furthermore, phytochemicals induce cytotoxicity in fungi by disrupting cell membrane permeability and functions; inhibiting cytoplasmic and mitochondrial enzymes; inhibiting enzymes involved in cell wall components synthesis; and altering the cell compartment, osmotic and the redox balance (Loi et al., 2020). However, plant extracts and their compounds also act by inducing xenobiotic detoxification and biotransformation pathways (Gross-Steinmeyer and Eaton, 2012; Wu et al., 2017b). Phytochemicals are capable of inhibiting enzymes that activate Phase I carcinogens as well as induce enzymes for Phase II detoxification (Galvano et al., 2001; Wu et al., 2017b). The bioactive compounds in plants have been used as additives to prevent fungal growth and aflatoxin (AF) contamination in food and feed (Table 1), thus reducing the risks of mutagenicity and carcinogenicity of such mycotoxins as AFs (Mathuria and Verma, 2007).

**Table 1:** Studies on the effects of plant extracts and their compounds on mycotoxin-induced toxicity.

Plant source	Protective agent	Model	Mechanism of action	References
**Native phytochemicals**				
*Curcuma longa L. (Turmeric)*	Curcumin	*In vitro* Ames assay, rodent & chicken	Hepatoprotective effects against AFB_1_ toxicity i*n vitro* and *in vivo*	Soni et al. (1997); Smerak et al. (2006a); Nayak and Sashidhar (2010); Limaye et al. (2018)
	Turmeric	Chicken broiler	Protected against AFB_1_ oxidative damage	Gowda et al. (2008); Nayak and Sashidhar (2010)
*Curcuma amada* (Ginger)	Ginger	HepG2 cells & rats	Inhibited AFB_1_, STE and PAT toxicity	Yang et al. (2011); Vipin et al. (2017)
	6-Gingerol	HepG2 cells *In vitro*	Reduced PAT induced DNA damage in HepG2	El-Shayeb (1980); Yang et al. (2011)
*Thymus vulgaris* (Thyme)	Thyme oil	Rats	Ameliorated oxidative stress and genotoxicity effects of AFB_1_	Abdel-Fattah et al. (2010)
*Syzygium aromaticum* (Clove)	Clove	*In vitro*	Inhibited AF, STE, & CIT toxicity	Hitokoto et al. (1980); Azzouz (1981); Hussain et al. (2012)
	Quercetin	*In vitro*	Prevented AFB_1_ carcinogenesis	Bhattacharya & Firozi (1988)
*Camellia sinensis L*. (green tea) and a variety of plants	Epigallocatechin-3- gallate (EGCG)	Mice	Protected against DON & HT-2 toxin toxicity	Smerak et al. (2006b); Yang et al. (2011); Do et al. (2015)
*Solanum lycopersicum* (tomatoes)	Lycopene	Chicken broiler, Mice & rats	Protected against oxidative, inflammatory, hormonal & reproductive damage induced by AFB_1_, OTA & ZEN in mice, also inhibited T-2 toxin induced oxidative stress	Leal et al. (1999); Aydin et al. (2013); Palabiyik et al. (2013); Wu et al. (2017a); Hedayati et al. (2018)
	Cyanidin	*In vitro* HepG2 & Caco-2 cells	Protected against AFB_1_ & OTA-induced oxidative stress	Sorrenti et al. (2012); Do et al. (2015)
	Rutin	Rats	Reversed T-2 toxin-induced lipid peroxidation in liver homogenate	El-sawi and Al-Seeni. (2009); Wu et al. (2017a)
**Crude extracts**				
*Premna integrifolia*		Mice	Inhibited AFB_1_ toxicity	Singh et al. (2019)
*Silybum marianu*		Broiler chicken	Inhibited OTA toxicity	Stoev et al. (2019)
*Withania somnifera*		Broiler chicken	Inhibited OTA toxicity *in vivo*	Stoev et al. (2019)
*Annona senegalensis*		*In vitro*	Inhibited AFB_1_ genotoxicity in Ames, Vitotox and comet assays	Makhuvele et al. (2018a)
*Monanthotaxis caffra*		*In vitro*	Inhibited AFB_1_ genotoxicity *in vitro*	Makhuvele et al. (2018b)

Antifungal and anti-mycotoxigenic activities of herbal plants with potential antioxidant properties were investigated against fungal strains that are phytopathogenic, i.e. *Fusarium verticillioides, A. flavus* and *A. ochraceous*. The results reported potentials of the selected medicinal plants to be used for the discovery of biofungicides that may prevent oxidative related food spoilage (Dikhoba et al., 2019). A study by Abdel-Fattah et al. (2018) reported the antioxidant, antifungal and anti-mycotoxigenic potentials of wild stevia extracts against *A. flavus, A. ochraceus, A. niger,* and *F. moniliforme.* Furthermore, essential oils have been found to effectively modulate the growth of mycotoxigenic fungi such as *A. favus, A. oryzae, A. niger, Alternaria alternata, F. moniliforme, F. graminearum, Penicillium citrinum* and *P. viridicatum*, etc., and their associated mycotoxins (da Cruz Cabral et al., 2013; Prakash et al., 2015; Powers et al., 2019). A study by Kocic-Tanackov et al. (2019) reported how essential oil of *Carum carvi L* applied at a concentration from 1.5 μl/g demonstrated complete inhibition of the growth of *A. parasiticus*, and likewise 4.5 μL/g exhibited complete inhibition of the growth of *A. flavus* as well as the secretion of aflatoxins by the same strain in polenta. Also noted was that a concentration of 35.0 μl/g of *Juniperus communis L.* essential oil showed strong potency against *A. parasiticus* and *A. flavus* IKB (i.e. by significantly inhibited their growth) with percentage inhibition between 42.4 and 79.8%, while 50.0 μl/g of *J. communis* impeded aflatoxin production by *A. flavus* IKB in polenta completely.

Curcumin and ellagic acid are examples of compounds isolated from plants which are used as food and feed supplements. These compounds prevent metabolism of aflatoxin B_1_ (AFB_1_) and increase the activity of glutathione-S-transferase involved in the detoxification of xenobiotics. They were equally found via Ames assay and in rat and chicken models to protect against mutagenicity induced by AFB_1_ with *Salmonella typhimurium* strain TA98 and TA100 (Soni et al., 1997; Gowda et al., 2008). Smerak et al. (2006a) studied the effect of curcumin against AFB_1_ mutagenicity by Ames, *in vivo* micronucleus, comet and chemiluminescence and blastic transformation methods. The results showed that curcumin was able to protect cells against mutagenicity. They also demonstrated a significant reduction in DNA damage through stimulation of DNA repairs. Curcumin has earlier been demonstrated by many authors to exhibit anticarcinogenic, antiproliferative and antimutagenic effects against various mutagens *in vitro* and *in vivo* (El-Hamss et al., 1999; Inano and Onoda, 2002; Polasa et al., 2004; Hosseini and Hosseinzadeh, 2018; Khan et al., 2019). Resveratrol is another natural product isolated from grape skin which suppresses the proliferation of many tumour cells such as breast, pancreatic and prostate cancers by inhibiting xenobiotic metabolism and inducing detoxification pathways (Farombi, 2004; Thipe et al., 2019). Studies have showed that this product can protect mycotoxin-induced toxicity in *vitro* and *in vivo* (Do et al., 2015; Sridhar et al., 2015; Tabeshpour et al., 2018). Furthermore, 6-gingerol, a natural bioactive compound extracted from ginger was found to possess strong protective effects against PAT-induced genotoxicity in HepG2 cells *in vitro* (Yang et al., 2011).

Lycopene is a natural product found in tomatoes, papaya and other red fruits and vegetables that showed to have protective effects against ZEN oxidative, reproductive and hormonal damage in mice (Aydin et al., 2013; Palabiyik et al., 2013). Lycopene also prevented T-2 toxin-induced oxidative stress and maintained GSH cellular levels *in vivo* (Leal et al., 1999). Furthermore, lycopene reduced AFB_1_ and OTA-induced oxidative stress and apoptosis in rats (Hedayati et al., 2018). Cyanidin, a phytochemical found in various medicinal herbs, fruits and vegetables including grapes, blackberry, cherry, cranberry, raspberry, red cabbage, red onion, etc., showed a protective effect against AFB_1_ and OTA-induced toxicity in hepatocytes and enterocytes (Sorrenti et al., 2012). El-Sawi and Al-Seeni (2009) reported that rutin displayed strong antioxidant activity against T-2 toxin in rat liver and also decreased lipid peroxidation induced by T-2 toxin. Shehata et al. (2017) demonstrated that oil-bioactive films from three extracts of immature fig fruit, leaves, and pomegranate husks to be a novel method for post-harvest grain management against mycotoxins.

Furthermore, Negera & Washe (2019) evaluated AF degradation abilities of some selected natural food spices including garlic, ginger, black cumin, clove, sacred basil, lemon grass, thyme, fenugreek and lemon, traditionally used by the Ethiopian community for food flavoring and preservation. Electrochemical and LC-MS/MS methods were used to investigate aflatoxin degradation efficacy of the spice extracts by determining the toxin in extract-treated and non-treated samples. The results revealed that garlic had maximum AFB_1_ degradation activity followed by lemon and the other dietary spices during 1-hour exposure to AFB_1_ standard at 25 °C. The results also showed that the possible mechanism of AFB_1_ degradation is through chemical transformation of AFB_1_ parent compound to another compound by the plant extracts. Iram et al. (2015) investigated the ability of *Corymbia citriodora* plant extract to detoxify AFB_1_ and AFB_2_ both *in vitro* and *in* v*ivo*. They observed that the leaf extracts had maximum detoxification at pH 8 and 30 °C temperature after 72 h of incubation. Iram et al. (2016) demonstrated that *Trachyspermum. ammi* seed extracts can be used for the development of biologically safe herbal additives in food and feed. Ponzilacqua et al. (2019) evaluated degradation capabilities of aqueous plant extracts of *Rosmarinus officinalis, Origanum vulgare, Psidium cattleianum* and *Passifora alata* against AFB_1_, with *R.* *officinalis* extract exhibiting the highest degradation percentage of AFB_1_ (range: 49.0–60.3%) at 24–48 h, followed by *O. vulgare* with (range of 30.7%–38.3%) after 48 h of incubation.

Makhuvele et al. (2018a) investigated the antigenotoxicity of plant extracts against AFB_1_-induced genotoxicity. The results showed that most plant extracts from *Artabotrys brachypetalus, Helichrysum petiolare, Hexalobus monopetalus, Friesodielsia obovate, Monanthotaxis caffra, Protea hybrid, Protea roupelliae, Monodora junodis, Uvaria caffra, Xylopia parviflora, Rhoicissus sekhukhuniensis, Podocarpus henkellii, Podocarpus elongatus* and *Agapanthus praecox* had moderate to strong antimutagenic potency in both Vitotox and Ames assays. Ethanolic extract of *Premna integrifolia* leaves demonstrated a protective effect in mice liver against AFB_1_-induced toxicity by inhibiting apoptosis and oxidative stress (Singh et al., 2019). Furthermore, extracts of *Schisandra chinensis* and *Thonninga sanguinea* were shown to possess hepatoprotective effects against AFB_1_ by enhancing antioxidant and detoxification in rats (Gyamfi and Aniya, 1998; Ip et al., 1999). Feed additives from South African medicinal plants, *i.e*., *Silybum marianum, Withania somnifera* and *Centella asiatica* were tested and except for *Centella asiatica*, the extracts were found to give partial protection against the growth inhibitory activity of OTA and associated immunosuppression in broiler chicks (Stoev et al., 2019). The same extracts also showed some hepatoprotective effects on broiler chicks exposed to OTA with extract from *S. marianum* exhibiting nephroprotective effect against OTA toxicity. Very recently, Nerilo et al. (2020) evaluated the use of ginger essential oils (GEO) as a fumigant agent for stored maize grains. They reported that GEO was mainly composed of α-zingiberene (23.85%) and geranial (14.16%). Furthermore, their results revealed that 25 and 50 μg/g, respectively, exhibited antifungal activity against *A. flavus* and inhibited AFB_1_ and AFB_2_ production. Despite the efficacy of plant extracts and their phytochemicals in the management of toxigenic fungi and their toxins, there are various limitations to the use of phytochemicals and their crude extracts as biofungicides and nutraceuticals. The subsequent section elaborates on these limitations.

### 2.4 Current limitations on the use of phytochemicals as biofungicide

There is an increased demand for the use of plant extracts and their compounds and this raises concerns about the safety, toxicity and quality of these products. Studies have reported the contamination of plant materials with medicinal properties by mycotoxins (Ashiq et al., 2014; Mulaudzi, 2019; Altyn and Twaruzek, 2020; Thipe et al., 2020). Furthermore, research has shown that many plant extracts used in traditional medicine and as food ingredients are toxic and mutagenic. As such, a thorough screening of their toxicological properties is necessary. Those that show no signs of toxicity are given high priority (Verschaeve and Van Staden, 2008; Sajid et al., 2019). The use of plant extracts, especially in exploiting their bioactive compounds, are exploited in the discovery and development of new antifungal and nutraceutical agents.

The high frequency of toxigenic fungal manifestation warrants the consistent evaluation of bioactive phytochemicals and their derivatives that exhibit high antifungal and anti-mycotoxigenic activities as alternatives to conventional fungicides. Table 2 briefly presents phytochemicals that have shown antifungal activities and their mode of action, thus inhibiting their respective mycotoxin biosynthesis. Moreover, nanoencapsulation formulations are also presented, exhibiting a synergistic effect between encapsulate and phytochemical with low side effects. The use of natural formulations antifungal agents for safe, effective, and eco-friendly features against fungi and mycotoxins is ideal for agricultural practices compared to standard antifungal agents (Rai et al., 2020). Literature precedence has proposed the notion of fully understanding the exact principles underlying the antifungal and anti-mycotoxigenic mode of action of phytochemicals against mycotoxigenic fungi (Chaudhari et al., 2020; Xu et al., 2020), facilitated through the following:

(i) inhibition of ergosterol biosynthesis, a major sterol that regulates plasma membrane biogenesis;
(ii) disruption of fungal cell membrane; and
(iii) production of reactive oxygen species (ROS) which results in oxidative stress.

**Table 2:** Antifungal effects of plant extracts, their compounds, and nanoformulations against mycotoxigenic fungi.

Plant source	Protective agent	Model	Mechanism of action	References
**Native phytochemicals**				
*Curcuma longa L. (Turmeric)*	Curcumin	*F. solani, C. albicans, P. expansum, A. flavus,* and *A. parasiticus*	Downregulation of Δ^5,6^ desaturase gene (*ERG3*) resulting in reduced ergosterol biosynthesis leads to cell death attributed by elevated levels of reactive oxygen species (ROS) production, Reduced proteinase secretion and inhibition of H^+^–ATPase activity induces acidification of extracellular and intracellular matrix inhibition of hyphae development through the suppression of thymidine uptake 1 (TUP1), Curcumin is a photosensitizer (0PS): Where 0PS + γ → singlet excited state (1PS∗) → triplet excited state (3PS∗) → H^+^ to a biomolecule = radicals (anion superoxide (O_2_^−^), hydrogen peroxide (H_2_O_2_), hydroxyl radical (•OH), and singlet oxygen (1O_2_) resulting in cell death by apoptosis, necrosis, or autophagy.	Sharma et *al.* (2010); Neelofar et al. (2011); Moghadamtousi et al. (2014); Chen et al. (2018); Song et al. (2020); Narayanan et al. (2020)
	Turmeric essential oil (e.g. *β*-pinene, camphor, and eucalyptol)	*A. flavus*	Fungicidal activity to destroy cellular integrity and induced alteration in mitochondrial membrane potential.	Hu et al. (2015)
*Curcuma amada* (Ginger)	α-zingiberene, geranial, 6-gingerol, and 6-shogaol	*A. flavus, A. parasiticus* and *F. verticillioides*	Anti-mycotoxinogenic activities through induced alteration in mitochondrial membrane potential which causes loss of membrane integrity, leakage of cellular material, and inhibition of ergosterol biosynthesis.	Kavitha et al. (2020); Nerilo et al. (2020); Gacem et al. (2020)
	Chitosan	*A*. *ochraceus*	Inhibition of gene expressions that are critical for cell wall and plasma membrane homeostasis, ribosome biogenesis and other biological processes through the distraction of the integrity of cell surface architecture and protein biosynthesis.	Meng et al. (2020)
*Rosmarinus officinalis* L. essential oil	1,8-cineole (eucalyptol), camphor and α-pinene	*A. flavus*	Reduces ergosterol production and biomass of mycelium.	da Silva Bomfim et al. (2019)
*Syzygium aromaticum* (Clove)	Eugenyl acetate, eugenol, and β-caryophyllene	*A. flavus* and *A. niger*	Induces cell death through early apoptosis (nuclear condensation) and late apoptosis (damage of plasma membrane) in hyphae, Downregulation of metabolic genes [secondary metabolism global regulator (*laeA*), lipase (*lipA*), and metalloprotease (*metP*)] responsible for fungal lipid and protein metabolism.	Oliveira et al. (2020); Castellanos et al. (2020)
*Punica granatum* (pomegranate)	Tannins	*Alternaria alternata, A. niger F. oxysporum*, *F. culmorum*, *F. graminearum*, and *P. digitatum*	Generation of ROS resulting in the destruction of the plasma membrane and mitochondrial dysfunction.	Zhu et al. (2019)
*Camellia sinensis L*. (green tea) and a variety of plants	Epigallocatechin 3-O-gallate (EGCG)	Candida *spp*.	Formation of lesions on the cell membrane caused by loss of cell membrane integrity, cellular and plasma membrane damage, Increased membrane permeability that causes osmotic imbalance which ultimately results in cell death.	Behbehani et al. (2019)
*Solanum lycopersicum* (tomatoes)	Lycopene	*C. albicans*	Plasma membrane depolarization and cell cycle arrest (G2/M) through increased intracellular ROS, Elevated levels of cytosolic and mitochondrial Ca^2+^ homeostasis causes mitochondrial dysfunction, Facilitates cytochrome *c* release that results in caspase activation.	Choi and Lee (2015)
*Piper nigrum* L. (Pepper)	limonene, sabinene, and *β*-caryophyllene	*F. oxysporum* and *A. niger.*	Disruption of cell wall and plasma membrane, coagulation of the cytoplasm, damage cellular organelles and ergosterol biosynthesis.	Castellanos et al. (2020)
**Nanoformulations**				
*Curcuma longa* L. and various fruits and vegetables	Nanovesicles (curcumin and quercetin co-encapsulion)	*A. niger, A. fumigates*, and Candida *spp*.,	Causes the excess formation of lomasomes and plasmalemmasomes which results in cell wall and plasma membrane expansion imbalance.	Sadeghi-Ghadi et al. (2020); Rai et al. (2020)
	Nanoemulsions (curcumin, piperine, and tualang honey) combinations	Candida *spp*.	Nanoemulsions (curcumin + piperine + honey) possessed favorable antifungal activity (more than 80%) against the wide range of *Candida* spp., due to multimodal activity.	Phuna et al. (2020)
*Streptomyces natalensis*	Natamycin/methyl-*β*-cyclodextrin (N/ME-*β*-CD)	*A. Japonicus, Gilbertella persicaria*, *Botrytis cinerea, and P. expansum*	Inhibits ergosterol biosynthesis in the plasma membrane resulting in mitochondrial dysfunction.	Kavitha et al. (2020); Yang et al. (2019)
*Origanum majorana* essential oil	Chitosan nanoemulsion	*A. flavus, A. parasiticus, F. graminearum*	Inhibits the production of ergosterol followed by release of cellular ions, Inhibition of methylglyoxal, *in situ* inhibition of lipid peroxidation.	Chaudhari et al. (2020)
Clove essential oil	Eugenol (EG) incorption into β-cyclodextrin (β-CD)	*Peronophythora litchii*	Causes hyphal and/or sporangiophore cell wall and plasma membrane damage leading to cell shrinkage, wrinkling, and partial distortion.	Gong et al. (2016)

The nanoencapsulation (e.g. nanoemulsion, nanofibre, nanogel, nanoliposome, nanoparticle, nanotube, and nanosponge) coupled with phytochemicals provides promising avenues to explore possible strategies for enhancing efficacy and combating fungal resistance where conventional antifungal drugs prove to be ineffective (Dhakar et al., 2019; Redondo-Blanco et al., 2019). Powers et al. (2019) emphasized on the priority and importance of big data approaches for expediting the identification of active components. They utilized a huge data to evaluate antifungal activity of 82 essential oils against *A. niger*, *C. albicans*, and *C. neoformans*. The results obtained demonstrated that antifungal susceptibility to the essential oils was as follows: *C. neoformans* > *C. albicans* > *A. Niger.* However, the use of phytochemical compounds alone limits their full applications. This limitation is due to their instability, insolubility, high volatility, low bioavailability, ability to change organoleptic characteristics of food/feed, and limited of facilities and resources for their extraction and purification (Sajid et al., 2019; Adebo et al., 2020; Loi et al., 2020). To overcome these factors, cohesive research collaborations between academics, research institutions, government agencies, food and pharmacological industries, as well as international stakeholders are required. In addition, different emerging technologies such as nanotechnology can, in part, provide solutions to encounter some of these limitations (Prakash et al., 2020; Thipe et al., 2020). Phytochemical compounds can be encapsulated in edible coatings or with nanoparticles such as nanosponges to elicit the factors listed herein (Alotaibi et al., 2019; Loi et al., 2020; Thipe et al., 2020). The subsequent section gives brief information on nanocarriers and their encapsulated bioactive compounds for use as antifungal and mycotoxin detoxification agents.

## 3 Innovative technology for mycotoxin detoxification

Frontiers in the field of nanotechnology have led to their vast applications in the field of nanomedicine, extending to the agricultural sector. Great strides have been made in the implementation of nanotechnology in agriculture for controlling mycotoxin contamination in food and feed supply chain (Thipe et al., 2018). The era of green nanotechnology, with the use of plant phytochemicals for the production of nanomaterials, has greatly improved their safety for use in agriculture as mycotoxin detoxifying agents (El-Desouky & Ammar, 2016; Thipe et al., 2020). A combination of nanotechnology and botanical extracts, as well as their phytochemicals, has shown significant results in the pharmacological, agricultural and cosmetic industries. Nano-encapsulated phytochemicals demonstrated strong efficacy over the free form because of the increased surface area, protection of encapsulated compounds from internal and external environmental conditions (Prakash et al., 2020). Nanocarriers can protect bioactive phytochemical compounds against thermal and photodegradation and further provide controlled release of antifungal compounds for the development of active packaging for maintaining the integrity of food/feed during storage and protection from fungal growth and mycotoxin contamination (Bahrami et al., 2020). They also caused reduced toxic effects of these plant-based drugs (Pushpalatha et al., 2018; Sajid et al., 2019). To date, there are different types of nanocarriers used for drug delivery, including liposomes, metal nanoparticles, polymeric nanoparticles, polymeric micelles and nanosponges. All these nanocarriers have been reported to be effective as drug delivery systems for plant-based products in cosmetics, agriculture and medicine (Pushpalatha et al., 2018; Thipe et al., 2020; Udomkun and Njukwe, 2020). However, there is limited information in the literature on the use of nanosponge encapsulated biofungicides, and for that, this paper highlights the use of cyclodextrin nanosponges as carrier vehicles to encapsulate phytochemicals.

### 3.1 Nanosponges as encapsulation system for antifungal and detoxifying phytochemicals

Nanosponges are emerging innovative drug delivery or encapsulation systems that result from the advancement of nanotechnology. They are microscopic, sponge-like particles with nanometer cavities, which can encapsulate both hydrophilic and lipophilic substances including toxins, but when doing so, enhance their stability, bioavalability and solubility (Ananya et al., 2020). Nanosponges are non-irritant, non-toxic, non-mutagenic and non-allergenic agents made-up of a polyester mixed with crosslinkers in a solution. The polyester is biodegradable and effective in delivering the drug to the targeted site (Pawar et al., 2016; Bhowmik et al., 2018). Nanosponges come in different forms including cyclodextrin which is most widely used and promising in encapsulation of phytochemicals. Cyclodextrin nanosponges are natural polymers formed from enzymatic degradation of starch and consist of a ring of oligosaccharides molecules. They are characterized by a highly porous spherical, amorphous or crystalline structure (Sherje et al., 2017). An interesting property of cyclodextrin-based nanosponges is that some important physicochemical properties of the nanomaterials such as dimension of the polymeric mesh, polarity and release of incorporated bioactive molecules can easily be manipulated by using different types of cross-linkers and varying the degree of cross-linking (Sherje et al., 2017; Pawar et al., 2019; Ananya et al., 2020). There are three widely known isomers of cyclodextrins, viz., alpha (⍺), beta (β) and gamma (ɣ) (Haimhoffer et al., 2019) that form inclusion and non-inclusion with different drugs to improve their stability, permeability, solubility and cytotoxicity (Osmani et al., 2014; Kumar et al., 2018).

Nanosponges can be used to mitigate challenges within the agricultural industry. For example, they can be used as biofungicides to replace synthetic pesticides currently used in agriculture to protect plants against pests and diseases for increased yield and quality of the agricultural products (Asghari et al., 2016). Nanosponges can be encapsulated with herbs that can be used for phytotoxicity problems on agricultural products. Asghari et al. (2016) reported the effects of nano-encapsulated herbicides against parasitic weeds. The herbicide encapsulated configuration allowed for the use of herbicides in different modes to selected areas since they were not affected (i.e. degraded) by the crop. The herbicides were applied in low doses and accumulated in the parasitic weed via the sink effect (Asghari et al., 2016). The use of nanosponges in mitigating fungal invasion and mycotoxin contamination can be crucial. Despite the beneficial properties of nanosponges and their wide applicability, very limited studies have investigated their use in mycotoxicology. Appell and Jackson (2012) investigated OTA sorption ability and characteristics of a β-cyclodextrin-based nanosponge (cyclodextrin-polyurethane polymer) in aqueous solutions. The results revealed that cyclodextrin nanosponge materials were effective at reducing OTA levels (between 1–10 μg/L) in spiked aqueous solutions. In fact, it was possible to reduce OTA levels from up to 10 μg/L in spiked red wine to within the recommended levels of 2 μg/L. Furthermore, inference from Langmuir isotherm of sorption data revealed that nanosponge has maximum binding capacity of 220 μg OTA per g of polymer. Elsewhere, solid phase extraction sorbent material based on a nanosponge was developed with β-cyclodextrin and methylene bis-diphenyl diisocyanate in the ratio of 1:5 (Appell et al., 2018). The novel polymer (i.e. β-cyclodextrin polyurethane polymer) was used to extract and clean-up OTA from wine and grape juice. Recoveries of OTA in the spiked beverages (0.5–20 ng/mL) as found, ranged from 69.1–86.5% in grape juice and 77.0–89.4% in wine (Appell et al., 2018). In another study, polyurethane-β-cyclodextrin polymers, in particular, polymer cross-linked with tolylene 2,4-diisocyanate were synthesized and the complex presented populations of binding sites which was able to extract patulin from apple juice (Appell and Jackson, 2010).

Researchers from Hungary investigated the pharmacoactive effects of β-cyclodextrin against the toxic effects of the xenoestrogenic mycotoxin, ZEN, in Tg (vtg1:mCherry) zebrafish embryos and HeLa cells. The results revealed that ZEN was able to form stable complexes with methyl-, sulfobutyl-, and succinyl-methyl-substituted β-cyclodextrins at pH 7.4 (K = 1.4–4.7 × 104 L/mol). The resultant complexes (*i.e*., modified cyclodextrins) strongly reduced or eliminated ZEN-induced mortality in zebrafish embryos and loss of cell viability in HeLa cells. Co-treatment with β-cyclodextrins further alleviated the sub-lethal effects of ZEN (Faisal et al., 2020). A study by Fliszar-Nyul et al. (2019) investigated association of alternariol toxin with γ- and β-CDs using molecular and fluorescence spectroscopy techniques. They also examined the extraction of the mycotoxin alternariol from water-based (aqueous) solutions using β-CD bead polymer (BBP) that is insoluble. The results demonstrated that natural γ-CD at pH 7.4 firmly increased the fluorescence of alternariol, which formed most stable complexes with natural γ-CD and quaternary ammonium derivatives under acidic/physiological pH and at pH of 10.0. Furthermore, BBP successfully extracted alternariol toxin from aqueous medium. The alternariol-binding capacity of BBP and β-CD polymers were strongly increased because of the β-CD content of the polymer. The effect of selective encapsulation of star anise essential oil (SAEO) by hydroxypropyl-β-cyclodextrin (HPCD) on its composition, stability, volatility, and antibacterial activity was investigated and results showed that encapsulation significantly decreased the irritating odour of SAEO and also improved the inhibitory effect of SAEO on *Saccharomyces cerevisiae, Rhizopus stolonoifer,* and *E. coli* and its antibacterial stability in 24 h (Zhang et al., 2018).

Studies have shown that natural products encapsulated in nanosponges yielded better results than with natural products alone (Ansari et al., 2011; Kumar et al., 2018). Nanosponges have been used as a stable carrier for the encapsulation of various therapeutic agents. Different types of phytochemical bioactive compounds such as resveratrol, curcumin, quercetin, rutin, oryzanol, chlorogenic acid, etc. have been encapsulated in cyclodextrin nanosponges (Kumar et al., 2018; Osmani et al., 2018; Pawar et al., 2019). A study by Ramírez-Ambrosi et al. (2014) evaluated the efficacy of β-cyclodextrin nanosponges for encapsulation of polyphenols phloridzin, rutin, and chlorogenic acid. The results showed that rutin had an encapsulation efficiency of 83.7 % (which was the highest) using 1,1′-carbonyldiimidazole as cross-linker in a 1:3 ratio of nanosponge/cross-linker, while phloridzin (87.2%) and chlorogenic acid (77.5 %), with best results seen with HMDI. Radic et al. (2020) evaluated the influence of olive pomace extracts matrix and cyclodextrins (CDs) on bio-accessibility and intestinal permeability of main olive pomace polyphenols. Their results demonstrated that olive pomace polyphenols were stable during gastrointestinal digestion and encapsulation of olive pomace polyphenol with cyclodextrins significantly increased bioaccessibility of tyrosol by forming inclusion complexes and preventing tyrosol adhesion to bile salts and other macromolecules present in reaction mixtures during simulation of olive pomace extract digestion. Despite the acclaimed pharmacological efficacy, there has been no investigation on cyclodextrin-based nanosponges encapsulation of phytochemicals for applications as biofungicides and nutraceuticals against mycotoxigenic fungi and mycotoxins. Nanosponges may play crucial role in the detoxification of mycotoxins in this era of the fourth industrial revolution (4IR) due to their binding and neutralization capacity (Fliszar-Nyul et al., 2019). This is because, these cyclodextrin-based nanosponges can reduce toxic effects associated with several microorganisms including mycotoxigenic fungi by binding and neutralising attendant harmful secondary metabolites in the body without leaving any harmful effects.

Cyclodextrins as nanosponges have been extensively used in a variety of applications, and a new class of them include integrated hydrogel cellulose nanosponges referred to as nanoemulgels that have recently attracted the attention of many scientists in the discovery and development of a variety of drugs. They are polymeric emulsion systems that provide synergistic complimentary properties from nanosponges or nanofibers and hydrogels, thereby reinforcing the overall antifungal activity in the food packaging sector (Oun et al., 2019; Munteanu and Vasile, 2020). Aldawasri et al. (2015) formulated lemongrass-loaded ethyl cellulose nanosponges with a topical hydrogel that forms a protective coating on the applied surface, whilst the encapsulated lemongrass oil had an enhanced antifungal activity against *C. albicans*. Bandyopadhyay et al. (2020) developed an effective green packaging technology for food safety and quality of gouda cheese. This technology was based extending the shelf life of gouda cheese using cinnamaldehyde and eugenol essential oils integrated to polyvinylpyrrolidone-carboxymethyl cellulose-bacterial cellulose-guar gum (PVP-CMC-BC-GG) hydrogel film (Munteanu and Vasile, 2020). The results revealed that this technology extended the shelf-life of cheese by more than 10–12 days, with a high antifungal activity against the tested *Aspergillus* and *Candida* species. Moreover, nanoemulgel formulations are ideal within the agricultural sector because they possessed repellent activities against insects. Lehtonen et al. (2020) demonstrated that addition of hexanal in aerogel extended the shelf life of plant-based products (*i.e*., blueberries and cherry tomatoes). The use of nanoemulgels in agriculture is still in its infancy but widely exploited in the medical sector. However, the popularity of nanoemulgels may likely increase as alternative antifungal agents in the agricultural sector. However, before that happens, it is imperative to conduct cycle analysis of any nanoformulation for agricultural applications so as to evaluate their overall ecotoxicology.

## 4 Conclusion and future perspectives

The search for alternative mycotoxin detoxification strategies is of paramount interest in food safety. From the literature reviewed herein, it was noted that the use of botanical extracts and their phytochemicals coupled with nanosponge encapsulation technology can reduce/detoxify mycotoxins with little or no negative consequences. Only a few studies have been carried out on the decontamination of mycotoxins using nano-encapsulated bioactive compounds. Cyclodextrin based-nanosponge encapsulated plant extracts or bioactive compounds can improve the efficacy of plant extracts or their phytochemicals for decontamination of mycotoxins as reviewed in this paper. This approach further increases the bioavailability of benign bioactive compounds utilized in agriculture as environmentally-friendly fungicides, highly-effective at low concentration with strong antifungal and mycotoxin-inhibiting activities.

Future work should seek to develop methods that may enable the use of these agents as food and feed additives for the same purpose. Detailed studies on the mechanisms of interaction between nano-encapsulated bioactive compounds and food components, together with their effects on human and animal health, should also be explored. Indeed, the potential of such nanosponges and plant bioactive compounds to play a dual pharmacological and nutraceutical function as biofungicides and detoxifying agents in mitigating the effects mycotoxins is very interesting.

## Declarations

### Author contribution statement

All authors listed have significantly contributed to the development and the writing of this article.

### Funding statement

This work was supported by the 10.13039/501100001321National Research Foundation (DSI/NRF), the joint LEAP-AGRI and NRF funding, and the 10.13039/501100006565University of Johannesburg, South Africa.

### Competing interest statement

The authors declare no conflict of interest.

### Additional information

No additional information is available for this paper.
